# Supplementary material for: Splice-Junction-Based Mapping of Alternative Isoforms in the Human Proteome
Source: Cell Rep. Author manuscript; Available in PMC 2020 Jan 15. (PMC6961840; doi:10.1016/j.celrep.2019.11.026)
Supplement: 2 [file NIHMS1546469-supplement-2.zip › DF1/PXD010154/Esophagus/Esophagus_33_PEX19_CFSHLSLLSGWDYS.pdf]

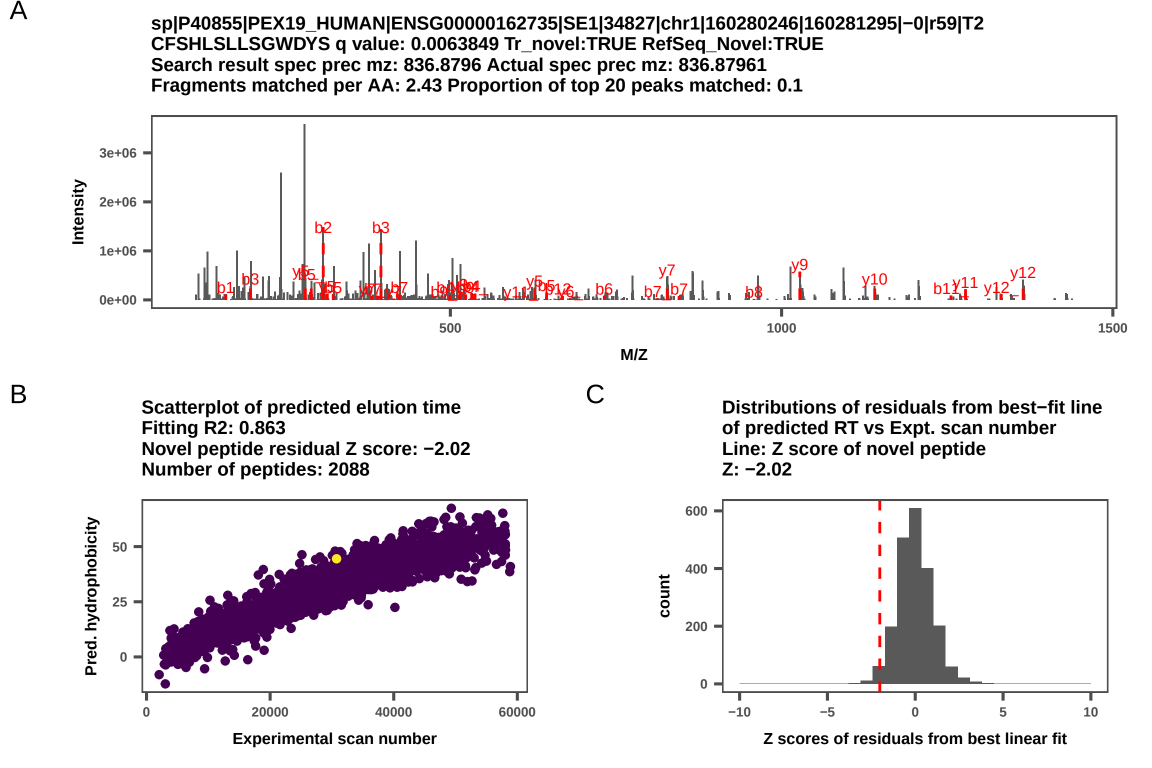
A
sp|P40855|PEX19_HUMAN|ENSG00000162735|SE1|34827|chr1|160280246|160281295|−0|r59|T2
CFSHLSLLSGWDYS q value: 0.0063849 Tr_novel:TRUE RefSeq_Novel:TRUE
Search result spec prec mz: 836.8796 Actual spec prec mz: 836.87961
Fragments matched per AA: 2.43 Proportion of top 20 peaks matched: 0.1
Intensity
3e+06
2e+06
1e+06
0e+00
b2
b3
y7
y9
y10
y12
b8
b6
M/Z
500
1000
1500
B
Scatterplot of predicted elution time
Fitting R2: 0.863
Novel peptide residual Z score: −2.02
Number of peptides: 2088
Pred. hydrophobicity
50
25
0
0
20000
40000
60000
Experimental scan number
C
Distributions of residuals from best−fit line
of predicted RT vs Expt. scan number
Line: Z score of novel peptide
Z: −2.02
count
600
400
200
0
−10
−5
0
5
10
Z scores of residuals from best linear fit
